# Reading Notices

**Published:** 1910-10

**Authors:** 


### READING NOTICES

### TONIC STIMULANT FOR THE HEART.

"Continued study, particularly of clinical cases, will show that Cactina Pillets is a mild tonic stimulant to the heart, acting both upon the nervous mechanism and directly upon the heart muscle. It promotes cardiac nutrition and overcomes atony of the heart muscle. Cactina has never been claimed to supplant digitalis, nor the other more powerful cardiac remedies. It has been shown, however, time and time again, that Cactina Pillets is especially serviceable in all functional disorders, as well as in certain phases of the common organic lesions."

### CONSTIPATION.

"Among the countless remedies that have been used for overcoming constipation, none have been found to possess qualifications so completely as Prunoids."

